# Supplementary material for: MacaquePose: A Novel “In the Wild” Macaque Monkey Pose Dataset for Markerless Motion Capture
Source: Front Behav Neurosci. 2021 Jan 18;14:581154. doi: 10.3389/fnbeh.2020.581154 (PMC7874091; doi:10.3389/fnbeh.2020.581154)
Supplement: Supplementary file 1 [file Image_1.PDF]

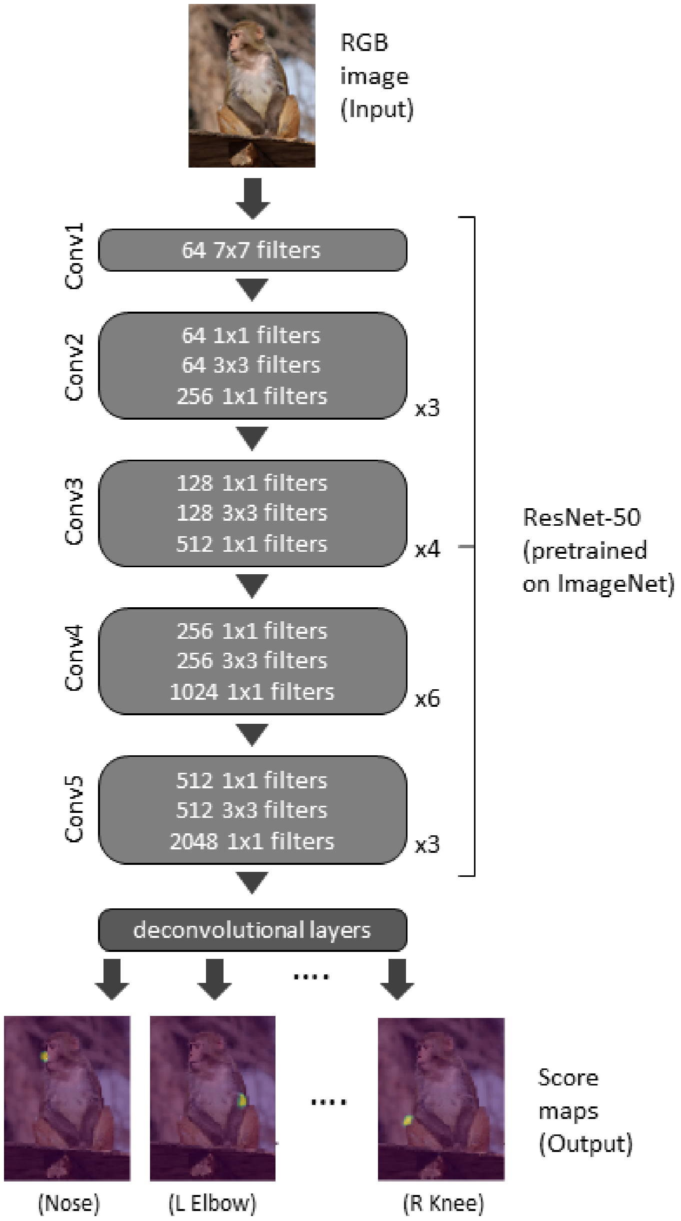

Supplementary Figure 1. Network architecture for monkey keypoint estimation using DeepLabCut algorithm. The network outputs 'score-maps' whose activity represents probability of corresponding keypoints.
