# Supplementary material for: MacaquePose: A Novel “In the Wild” Macaque Monkey Pose Dataset for Markerless Motion Capture
Source: Front Behav Neurosci. 2021 Jan 18;14:581154. doi: 10.3389/fnbeh.2020.581154 (PMC7874091; doi:10.3389/fnbeh.2020.581154)
Supplement: Supplementary file 2 [file Image_2.PDF]

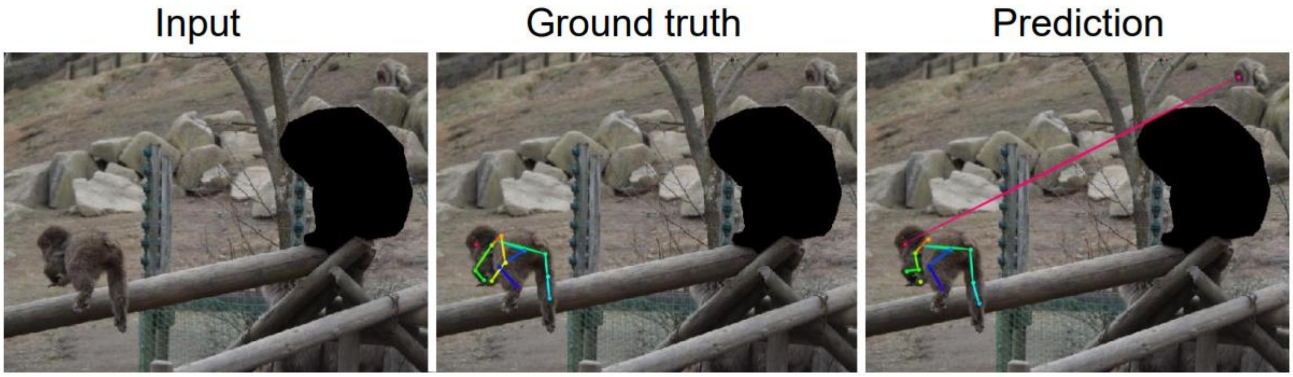

Supplementary Figure 2. An example of ‘out of monkey’ keypoint detection error. Keypoint detection error due to the imperfect masking of the other monkey.s
