# Supplementary material for: MacaquePose: A Novel “In the Wild” Macaque Monkey Pose Dataset for Markerless Motion Capture
Source: Front Behav Neurosci. 2021 Jan 18;14:581154. doi: 10.3389/fnbeh.2020.581154 (PMC7874091; doi:10.3389/fnbeh.2020.581154)
Supplement: Supplementary file 3 [file Image_3.PDF]

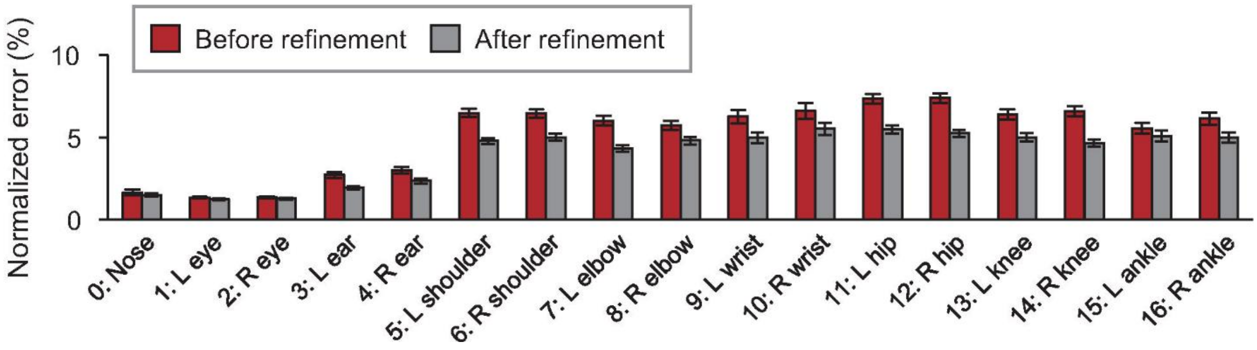

Supplementary Figure 3. Averaged error observed before and after the researchers' refinement. Averaged error for prediction by the network trained with the dataset before (red) and after (gray) the refinement by researchers. Error bars represent standard error of the mean.
